# Aural Myiasis, a Rare Cause of Earache

**DOI:** 10.1155/2015/219529

**Published:** 2015-08-24

**Authors:** Ibrahim Al Jabr

**Affiliations:** Department of Otolaryngology and Head and Neck Surgery, College of Medicine, King Faisal University, P.O. Box 66678, Al-Ahsa 31982, Saudi Arabia

## Abstract

Myiasis of the ear is an infestation of the ear by maggots (the larval stage of flies). In the literature, there are only few cases reported about aural myiasis. It is more common to occur in tropical regions, where humidity and warm weather provide a good environment for this infestation. In this paper, a 12-year-old boy is reported to have unilateral earache for 3-day duration. Examination of the painful ear showed a tympanic membrane perforation with larvae (maggots) in the middle ear. They were removed by using a forceps and gentle irrigation of ear to expel any remnant. Further management included assessment of hearing, computed tomography (CT) scan, and outpatient follow-up.

## 1. Introduction

Myiasis is a common infestation among mammals. In humans, it is seen more in rural areas where people are in more direct contact with animals [1]. The disease occurs when the female fly lays eggs, which shortly will cause clinical manifestations that are related to the body site involved [2]. In the field of otolaryngology, it may affect the ears, nose and paranasal sinuses, nasopharynx, oral cavity, and skin of the head and neck region. Risk factors for myiasis in humans are chronic suppurative otitis media, low socioeconomic status, swimming in stagnant water, and diabetes mellitus [3]. Other possible predisposing factors include neglected children, old age, mental retardation, and poor personal hygiene.

## 2. Case Report

A 12-year-old boy, previously healthy, presented to the emergency department complaining of right earache for 3-day duration. This ear pain started suddenly and the patient has described it as being mild to moderate in severity. The patient is complaining also of minor decrease in hearing and itching in the ear canal. There was no history of purulent, bloody, or clear ear discharge, tinnitus, vertigo, or facial weakness. There was no history suggestive of intracranial involvement. Social history showed that the patient lives in dessert with his family, in a Bedouin culture.

**Figure 1:**
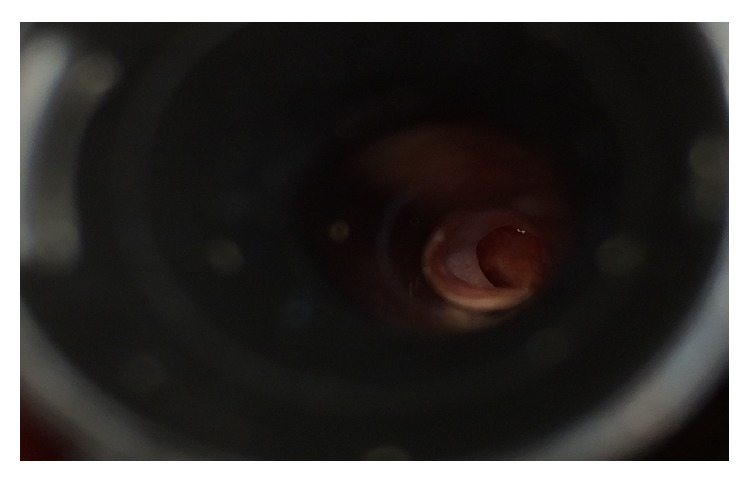
Microscopic view of the right ear, showing a clean central perforation of the tympanic membrane after the removal of the larvae.

**Figure 2:**
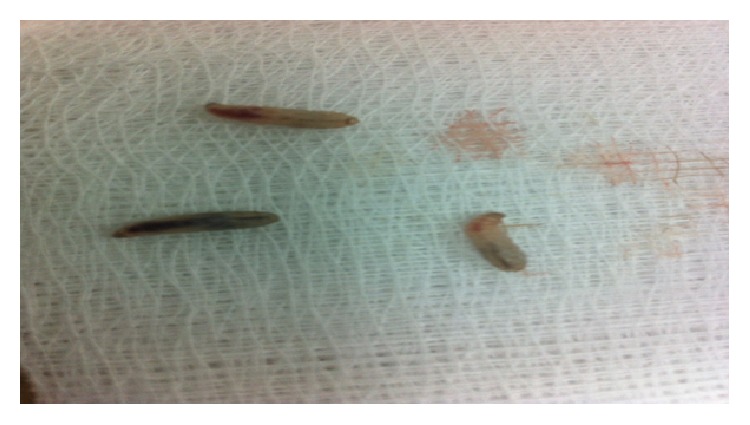
Three alive larvae removed from the patient ear.

General clinical examination, vital signs, and examination of nose, throat, left ear, head, and neck were all within normal. Inspection of the right ear externally was unremarkable; there is mild to moderate tenderness with pressure over the tragus or by gentle movement of the auricle. Examination by otoscope and microscope showed mild edema and erythema of the external auditory canal, a clean central perforation of the tympanic membrane of about 5 mm (Figure 1), and 2 larvae in the middle ear, protruding through the perforation. A crocodile forceps was used to remove these 2 larvae (Figure 2). Irrigation of the ear with sterile water was done, after which 4 more larvae which were in the attic region and not visible showed up and came out to the external auditory canal through the perforation and were removed. Further carful inspection and irrigation were made but did not show any more remnants. The removed larvae were identified by microbiologist to belong to the Sarcophagidae family, genus* Wohlfahrtia*, and species* Wohlfahrtia magnifica*. It is also known as spotted flesh fly or Wohlfahrt's wound myiasis fly. The larvae are cylindrical in shape with flattened ventral surface, 8 to 12 mm in length, white in color with grayish tinge. Spines are separating the body segments of the larvae. They have the characteristic sarcophagid posterior end, with the posterior spiracles set in a cavity.

The patient was admitted to the ward overnight for further management. He was started on mild analgesics and prophylactic antibiotic treatment to prevent possible secondary infections. On the second day of admission, reexamination showed improvement of the pain, edema, and erythema and no more larvae in the ear.

The patient underwent audiological assessment (tympanometry and pure tone audiogram), which showed flat tympanogram (type B) and mild conductive hearing loss in the involved ear. Also he underwent CT scan to rule out any intracranial involvement, and it showed normal middle ear and intracranial structures and intact roof for middle ear and mastoid.

After that the patient was discharged, and outpatient follow-up was arranged for him. After 3 weeks of the discharge, the perforation healed completely, and the repeated audiological assessments were within normal.

## 3. Discussion

Aural myiasis is a rare infestation of the ears. According to a recent published review article, there are only 45 reported cases of aural myiasis [4]. Myiasis can be classified into either obligatory or facultative infestation. In the former, the host, most commonly the goat and sheep, is an obligatory part of the life cycle of the maggots, while in the latter it is not [5]. The infestation found in this patient (Sarcophagidae family,* Wohlfahrtia magnifica* species) is an obligate parasite. The female fly is attracted to normal and pathological secretions of the orifices of mammals [6].

Patients usually present to the hospital complaining of ear pain, hearing loss, purulent or bloody ear discharge, itching in the ear, and/or tinnitus [7–9]. Other possible presentations may include vertigo, facial weakness, and/or neurological manifestations secondary to intracranial involvement. The symptoms start after the deposited larvae start to feed on the surrounding tissues. The infestation is usually diagnosed by history and clinical examination, which will show the larvae in the ear. It is less likely to need further investigations to diagnose it, because the larvae are usually present near the external auditory canal because they need air for breathing.

The treatment for aural myiasis is usually simple in most of the cases, requiring nothing more than removal of the larvae and irrigation of the ear by one or more of the following solutions: alcohol, chloroform, normal saline, oil, ivermectin, or iodine [7–9]. Also, prophylactic broad spectrum antibiotics are usually prescribed to prevent secondary infections.

The larvae should be removed under microscope with careful inspection for any residual. The best choice for irrigation solution is debatable as all of them achieve the same outcome. The goal of the irrigation is usually to kill and expel any residual larvae, mainly the ones not visible or accessible on examination.

Surgical exploration is sometimes needed in patients when there is suspicion about the extent of the disease or for residual disease. In these cases, usually mastoid exploration is performed and the extent of the infestation is identified and if any residual is found it will be removed [10, 11]. In the case reported here, there was no suspicion of any residual disease and there were no manifestations that may raise the suspension of intracranial extension. Also, CT scan showed intact bony landmarks and normal intracranial space, with no suspicion of any residual disease. We suggest that if a patient is going to have surgical exploration, CT scan should be done before. It may obviate the need for the surgical intervention, especially in patients with low suspicion, and if not it will be useful as a preoperative preparation and assessment for surgical landmarks.

Management of these patients should also include hearing assessment to document any change in the hearing level and for future comparison.

Intracranial extension, at least theoretically, is a possible dangerous complication of aural myiasis. A review of the 45 reported cases diagnosed to have aural myiasis did not show any intracranial involvement secondary to an infested ear [4]. Intracranial involvement of patients with aural myiasis must be looked after carefully in all patients, especially in the presence of manifestations that may raise suspicion, for example, clear otorrhea, headache, or seizure.

In conclusion, aural myiasis is a rare infestation of the ear. It occurs usually in patients with risk factors like chronic suppurative otitis media, low socioeconomic status, neglected children, old age, mental retardation, and poor personal hygiene. The clinical presentation may range from mild earache to manifestation of intracranial extension like seizure. Treatment is usually simple, by removal of the larvae, ear irrigation, and antibiotics to prevent any possible secondary infection.
